# Ultrathin BiO_*x*_ Charge-Transfer Doping for Cross-TMD Electronic Modulation and Homogeneous WSe_2_ Inverters

**DOI:** 10.1021/acs.nanolett.6c03585

**Published:** 2026-10-19

**Authors:** Ching-Hao Hsu, Yu-Wei Hsu, Ta-Wei Hsu, Yu-Tung Lin, Li-Ya Chiu, I-Chih Ni, Chih-I Wu

**Affiliations:** † Graduate Institute of Photonics and Optoelectronics, National Taiwan University, Taipei 106, Taiwan; ‡ Graduate School of Advanced Technology, 33561 National Taiwan University, Taipei 106, Taiwan

**Keywords:** bismuth oxide (BiO_*x*_), charge-transfer doping, contact engineering, field-effect transistors (FETs), inverter, threshold-voltage engineering, transition-metal dichalcogenides (TMDs), two-dimensional semiconductors

## Abstract

Charge-transfer doping provides a nondestructive route to tune two-dimensional transition-metal dichalcogenides (TMDs), but selectively patterned n-type dopants effective across different TMD channels remain limited. Here, ultrathin bismuth oxide (BiO_*x*_) is used as an n-type charge-transfer doping and passivation layer for monolayer MoS_2_ and WSe_2_ transistors. In MoS_2_, 2 nm BiO_*x*_ preserves the channel structure and yields a TLM-extracted effective contact resistance of 0.71 ± 0.06 kΩ·μm at n_2D_ = 1.8 × 10^13^ cm^–2^. Thicker BiO_*x*_ strengthens n-type modulation but reduces gate controllability. The modulation remains stable after ambient storage and air annealing. BiO_*x*_ also enhances electron transport in WSe_2_ and enables homogeneous WSe_2_ inverters with MoO_*x*_-doped p-FETs, achieving a gain of ∼19 and a total normalized noise margin of 0.895 V_DD_ at V_DD_ = 2 V. These results establish BiO_*x*_ as a cross-TMD n-type charge-transfer dopant for contact, threshold-voltage, and polarity engineering.

## Graphical abstract

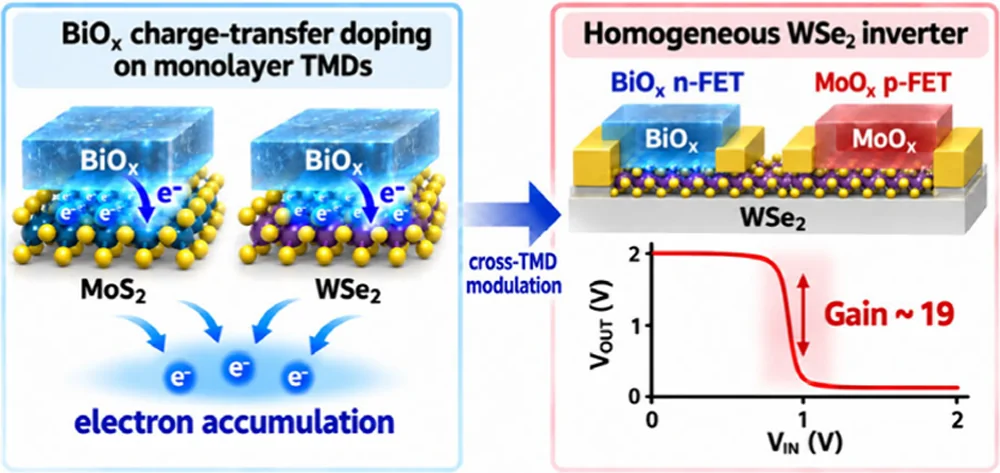

Monolayer transition metal dichalcogenides (TMDs) are promising channel materials for scaled field-effect transistors. Their atomic thickness enables strong gate control, and their van der Waals surfaces help suppress dangling bond-related interface states during device integration.^1–5^ These features make TMDs attractive for ultrathin transistors that require integration with metal contacts, gate dielectrics, and interfacial doping layers. However, favorable electrostatics alone do not guarantee logic-level operation. Practical TMD circuits require low contact resistance, controllable threshold voltage (V_TH_), stable polarity control, and environmental robustness.^6–8^ These requirements are closely coupled. For example, a contact scheme that increases the on-state current may not provide the desired V_TH_, whereas a doping layer that shifts V_TH_ may also increase the off-state leakage or degrade long-term stability. In TMD inverter circuits, the n- and p-type branches must also be properly balanced. A weak n-type device, excessive doping, or an unstable interface can shift the switching voltage, lower the voltage gain, and reduce the noise margin.^9–12^ These coupled constraints make carrier density and interface control central challenges for monolayer TMD electronics.

Conventional doping approaches are difficult to apply to monolayer TMDs. Ion implantation and substitutional incorporation can introduce vacancies, lattice disorder, and irreversible defect states, because the entire channel consists of only a few atomic planes. Chemical functionalization, molecular dopants, and intercalants can produce large carrier-density or polarity changes, but many of these schemes suffer from desorption, spatial nonuniformity, environmental sensitivity, or impurity scattering.^13,14^ Charge-transfer doping (CTD) offers a less invasive route because the channel is modulated by interfacial energy-level alignment and charge redistribution rather than by replacing atoms in the lattice.^15,16^ Among CTD approaches, oxide-based layers are particularly relevant for device integration because their thickness, stoichiometry, work function, and defect states can be engineered by using thin-film deposition. Prior oxygen-deficient high-k oxides and substoichiometric TiO_*x*_ layers have demonstrated n-type modulation of monolayer MoS_2_ through interfacial electron transfer and oxide-related donor states.^15,16^ These reports establish oxide CTDs as an important direction, but they also set a higher standard for new dopant layers. The advancement should not rely only on a V_TH_ shift in a single device structure. Instead, an effective oxide dopant should preserve the monolayer channel, be compatible with selective patterning, and provide controllable contact and V_TH_ modulation across different TMDs.

In this work, ultrathin bismuth oxide (BiO_*x*_) is investigated as an n-type charge transfer doping and passivation layer that can address these coupled requirements. Monolayer MoS_2_ FETs are first used as a representative n-type TMD transistor platform. Au contacts are intentionally adopted to avoid masking the BiO_*x*_-induced contact modulation with other aggressive contact-engineering schemes, allowing the contribution of BiO_*x*_ to carrier injection and contact resistance reduction to be directly evaluated.^17,18^ The same BiO_*x*_ layer is then extended to WSe_2_, whose ambipolar transport characteristics make it suitable for homogeneous inverter applications, with the specific goal of achieving controlled n-type modulation of the WSe_2_ branch.^12,19,20^ Finally, BiO_*x*_-doped WSe_2_ n-FETs are paired with MoO_*x*_-doped WSe_2_ p-FETs to form homogeneous WSe_2_ inverters, providing a circuit-level test of whether BiO_*x*_-induced n-type modulation can be translated into a balanced pull-down operation.

To clarify the role of BiO_*x*_, multiple materials and interfacial and electrical characteristics are examined. AFM and cross-sectional TEM were used to examine the surface morphology and cross-sectional ultrathin overlayer structure on monolayer MoS_2_. Raman and photoluminescence spectroscopies are employed to assess whether the TMD lattice and optical response are preserved after BiO_*x*_ deposition. X-ray photoelectron spectroscopy was used to analyze the chemical states at the BiO_*x*_/TMD interface and to examine whether destructive chemical reactions occur. Ultraviolet photoelectron spectroscopy is further used to track the TMD-side effective work function and valence-band shifts associated with BiO_*x*_-induced electronic redistribution. These analyses are compared with the electrical characteristics to relate the interfacial electronic modulation to the observed V_TH_ shift and carrier injection improvement.

Monolayer MoS_2_ FETs were first fabricated on TiN/8 nm HfO_*x*_ back-gate substrates to evaluate the structural compatibility of BiO_*x*_ capping. Figure 1a shows the device structure, in which Au source/drain contacts define the MoS_2_ channel and an ultrathin BiO_*x*_ layer selectively covers the active region. The optical image in Figure 1b confirms that BiO_*x*_ is selectively deposited on the device area. The AFM image in Figure 1c shows that the BiO_*x*_-capped surface prepared from a nominal 2 nm Bi precursor layer is continuous and smooth, with a root-mean-square roughness of approximately 0.5 nm. Cross-sectional TEM in Figure 1d further reveals an ultrathin overlayer at the expected position of the BiO_*x*_ capping layer above the MoS_2_/HfO_*x*_ stack. These observations are important, because CTD relies on intimate interfacial contact between the dopant layer and the monolayer channel. If BiO_*x*_ formed discontinuous islands or a highly rough overlayer, then the resulting electrical changes would be difficult to assign to a well-defined interfacial doping effect. The continuous ultrathin morphology provides the intimate BiO_*x*_/MoS_2_ interface required for the spectroscopic and electrical analyses discussed below.

**Figure 1:**
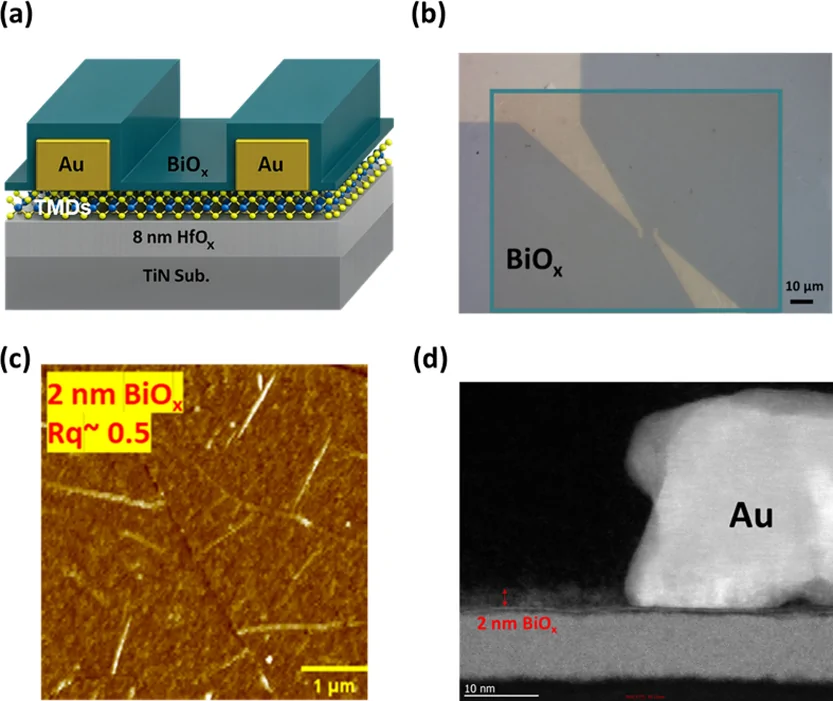
Device structure and morphology of BiO_*x*_-capped monolayer MoS_2_ FETs. (a) Schematic illustration of the back-gated monolayer MoS_2_ FET with Au source/drain contacts and an ultrathin BiO_*x*_ capping layer on an 8 nm HfO_*x*_/TiN gate stack. (b) Optical microscopy image of a representative BiO_*x*_-capped MoS_2_ device, where the BiO_*x*_-covered region is marked by the colored frame. (c) AFM image of a BiO_*x*_-capped monolayer MoS_2_ sample prepared from a nominal 2 nm Bi precursor layer, showing a root-mean-square roughness of approximately 0.5 nm. (d) Cross-sectional TEM image of the nominal BiO_*x*_/Au/MoS_2_ stack, showing the morphology of the ultrathin capping layer above monolayer MoS_2_.

Spectroscopic characterization was then performed to correlate the BiO_*x*_-induced doping effect with the interfacial and structural properties of MoS_2_. Figure 2a shows the Raman spectra of monolayer MoS_2_ capped with 2, 4, and 6 nm BiO_*x*_. The characteristic in-plane E_2g_ and out-of-plane A_1g_ vibrational modes are retained after BiO_*x*_ deposition, indicating that the monolayer lattice is not severely degraded by the capping process.^21^ More importantly, the A_1g_ mode exhibits a doping-related shift, while the E_2g_ mode remains comparatively less affected. This behavior is consistent with the known sensitivity of the A_1g_ phonon to electron doping in monolayer MoS_2_, where charge-induced electron–phonon coupling selectively modulates the out-of-plane vibration.^22^ The absence of pronounced peak broadening or additional disorder-related Raman features further supports the structural compatibility of the BiO_*x*_ overlayer. Figure 2b shows the corresponding photoluminescence spectra. After BiO_*x*_ capping, the PL intensity is suppressed and the emission profile is modified. This PL quenching is consistent with n-type charge-transfer doping of monolayer MoS_2_.^23,24^ Previous chemical-doping studies have shown that electron-donating dopants reduce the PL intensity of 1L-MoS_2_, whereas p-type dopants enhance it, because the injected carriers change the balance between neutral exciton and charged-trion emission. In doped monolayer MoS_2_, negative trions composed of two electrons and one hole have also been spectroscopically identified, further supporting the sensitivity of the PL response to electron-density modulation.^25^ Therefore, the reduced PL intensity after BiO_*x*_ deposition is consistent with increased electron density in MoS_2_ and the associated redistribution of excitonic recombination pathways.

**Figure 2:**
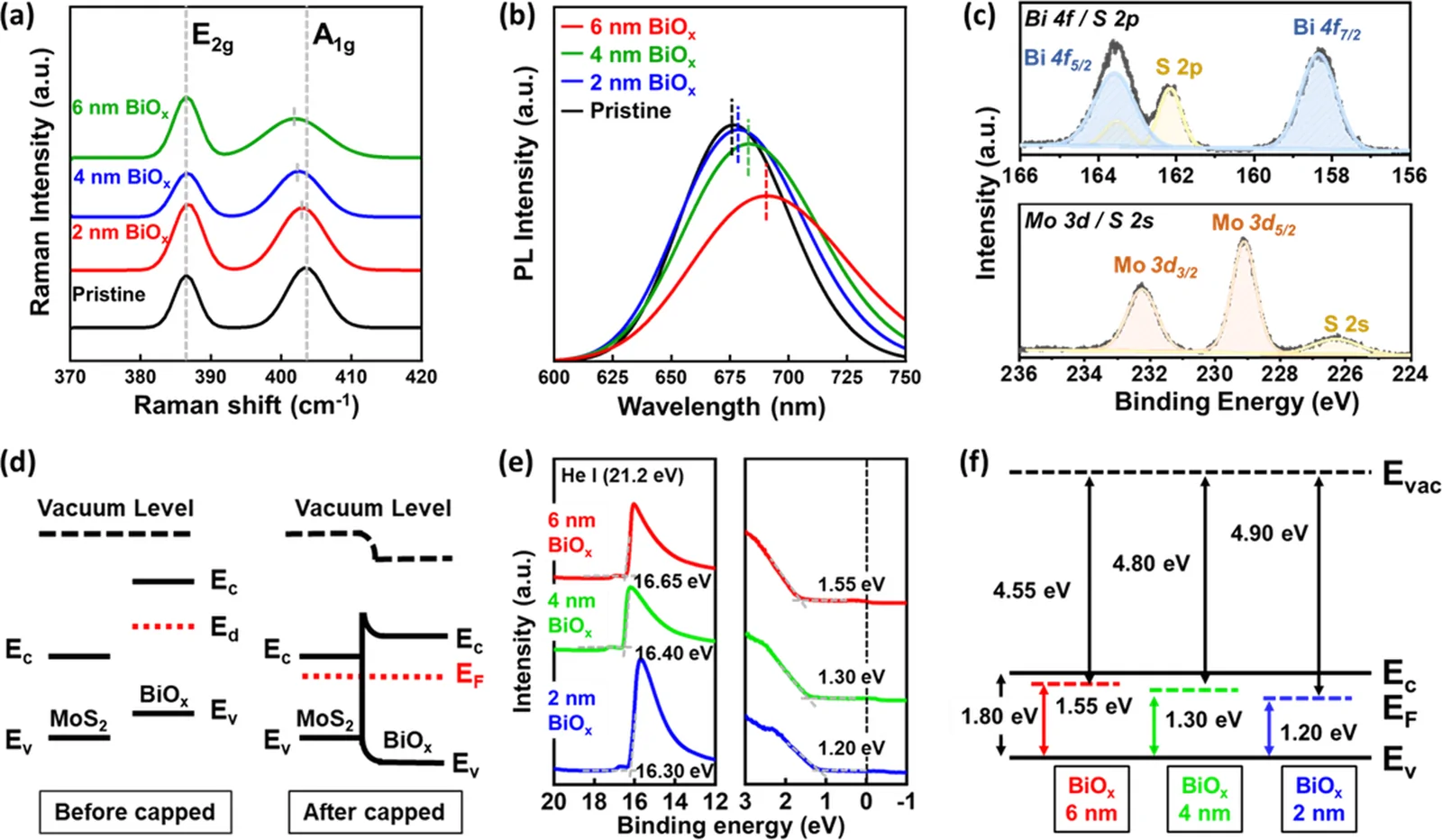
Spectroscopic and energy-level characterization of BiO_*x*_-capped monolayer MoS_2_. (a) Raman spectra of pristine monolayer MoS_2_ and samples capped with 2, 4, and 6 nm BiO_*x*_. (b) Photoluminescence spectra of pristine and BiO_*x*_-capped monolayer MoS_2_ with different BiO_*x*_ thicknesses. (c) XPS spectra of Bi *4f*/S *2p* and Mo *3d*/S *2s* core levels for BiO_*x*_-capped MoS_2_. (d) Qualitative schematic illustrating the proposed interfacial electron-transfer and charge-redistribution process upon electronic equilibration of the BiO_*x*_/MoS_2_ heterostructure. The BiO_*x*_ energy levels are schematic and are not intended to represent a directly measured standalone BiO_*x*_ work function. (e) TMD-side UPS secondary-electron cutoff and valence-band spectra of backside-exposed BiO_*x*_/MoS_2_ heterostructures prepared with nominal 2, 4, and 6 nm Bi. The measurements were performed directly from the exposed MoS_2_ side after inverted transfer and removal of the sapphire growth substrate. (f) TMD-side energy-level alignment of the BiO_*x*_/MoS_2_ heterostructure derived from backside UPS measurements, showing progressive Fermi-level modulation toward the MoS_2_ conduction-band edge with increasing nominal Bi thickness. The diagram represents the TMD-side energy-level evolution and does not constitute a direct measurement of the Au/MoS_2_ Schottky barrier height. The extracted work functions correspond to the exposed TMD side of the equilibrated BiO_*x*_/MoS_2_ heterostructure and should not be interpreted as standalone BiO_*x*_ work functions.

XPS was further used to examine the chemical states at the BiO_*x*_/MoS_2_ interface, as shown in Figure 2c. The Bi *4f* core-level spectra confirm the oxidized Bi states in the BiO_*x*_ capping layer, while the Mo *3d*, S *2p*, and S *2s* signals from MoS_2_ remain clearly identifiable after BiO_*x*_ deposition. No additional bonding components associated with strong Bi–S or Bi–Mo reactions are observed within the detection limit of the measurement. This result indicates that BiO_*x*_ capping does not chemically consume or convert the MoS_2_ channel, supporting a nondestructive interfacial modulation process. Quantitative XPS analysis further reveals that the ambient-oxidized BiO_*x*_ layers are substoichiometric. The sensitivity-factor-corrected O/Bi atomic ratios are approximately 1.3, 1.1, and 1.0 for samples prepared from nominally 2, 4, and 6 nm Bi layers, respectively, all below the stoichiometric O/Bi ratio of 1.5 for Bi_2_O_3_ (Figure S1). The decreasing O/Bi ratio with increasing nominal Bi thickness indicates progressively incomplete ambient oxidation and a more oxygen-deficient composition. Consistently, the Bi 4f spectra are dominated by oxidized Bi–O species for all thicknesses, whereas the nominal 6 nm sample additionally exhibits a weak low-binding-energy Bi component, suggesting a minor residual metallic/reduced Bi contribution. The presence of this weak reduced-Bi component does not by itself establish the formation of a laterally continuous metallic conduction path across the transistor channel. No clearly resolved metallic-Bi contribution is observed for the 2 and 4 nm samples. These results establish the thickness-dependent chemical composition of the BiO_*x*_ overlayer. Based on these spectroscopic observations, Figure 2d qualitatively illustrates the proposed interfacial electron-transfer and charge-redistribution process upon electronic equilibration of the BiO_*x*_/MoS_2_ heterostructure. This electronic redistribution drives the MoS_2_ Fermi level toward the conduction-band edge without requiring substitutional doping of the TMD lattice. UPS measurements were performed directly from the exposed backside of monolayer MoS_2_ using the inverted sample geometry described in the Supporting Information (Figure S6). As shown in Figure 2e, the secondary-electron cutoff shifts from 16.30 to 16.40 and 16.65 eV for samples prepared with nominal 2, 4, and 6 nm Bi, respectively. Using the He I photon energy of 21.2 eV, the corresponding TMD-side effective work functions are extracted to be 4.90, 4.80, and 4.55 eV. Because the photoemission surface is the exposed MoS_2_ rather than the BiO_*x*_ overlayer, these changes reflect the evolution of the electronic potential at the MoS_2_ side of the equilibrated BiO_*x*_/MoS_2_ heterostructure. The valence-band spectra simultaneously show that the energy separation between the Fermi level and the valence-band maximum increases from 1.20 to 1.30 and 1.55 eV with increasing nominal Bi thickness.^26,27^ Considering the monolayer MoS_2_ bandgap of approximately 1.80 eV,^28^ these results indicate progressive movement of the MoS_2_ Fermi level toward the conduction-band edge, as summarized in Figure 2f.^29^ These UPS-derived quantities characterize the TMD-side energy-level modulation and are not used here to determine the Schottky barrier height at the buried Au/MoS_2_ interface.

The observed n-type modulation is attributed to interfacial electron transfer and charge redistribution upon electronic equilibration of the BiO_*x*_/MoS_2_ heterostructure. The backside UPS measurements directly establish the resulting TMD-side Fermi-level modulation but do not provide the standalone work function of the BiO_*x*_ overlayer. Published studies show that the electronic properties and work function of Bi_2_O_3_/BiO_*x*_ are strongly dependent on phase, stoichiometry, surface condition, and processing. Direct UPS measurements of β-Bi_2_O_3_, for example, have reported a work function of approximately 4.73 eV, while nanometer-scale Bi_2_O_3_ surface-modification layers have been shown to produce effective electrode work functions near 4.22 eV.^30,31^

Quantitative XPS further confirms that the ambient-oxidized BiO_*x*_ in the present study is substoichiometric. This defect-rich/reduced composition may modify the electronic structure of BiO_*x*_ and contribute to its electron-donating character. Notably, ambient-grown oxygen-deficient Bi_2_O_3−δ_ with an O/Bi ratio of approximately 1.34 has been reported to exhibit occupied defect-related states near the Fermi level, while calculations indicate that oxygen vacancies shift the Fermi level toward the vacuum level relative to vacancy-free Bi_2_O_3_.^32^ However, because the absolute BiO_*x*_ work function is strongly preparation dependent and the higher-binding-energy O 1s components are not unique signatures of oxygen vacancies, we do not assign a literature-derived work function to our BiO_*x*_ films or identify oxygen vacancies as the exclusive microscopic donor species. Instead, interfacial electronic equilibration and the experimentally observed TMD-side Fermi-level shift are taken as direct evidence for BiO_*x*_-induced n-type charge-transfer modulation.

The transistor-level impact of BiO_*x*_ capping was then examined using monolayer MoS_2_ FETs. As shown in Figure 3a, the transfer characteristics measured at V_D_ = 1 V exhibit a clear negative V_TH_ shift after BiO_*x*_ deposition, indicating increased electron density in the MoS_2_ channel. The modulation is thickness dependent: 2 nm BiO_*x*_ produces a moderate n-type shift while retaining clear gate modulation, whereas 6 nm BiO_*x*_ induces substantially stronger electron accumulation and a higher drain current but also substantially increases the off-state current. The elevated off-state current is primarily attributed to incomplete electrostatic depletion of the strongly n-doped MoS_2_ channel within the experimentally accessible gate-voltage range. This interpretation is consistent with the BiO_*x*_-thickness-dependent Fermi-level movement toward the conduction-band edge observed by backside UPS. Although XPS reveals a minor residual metallic/reduced Bi component in the nominal 6 nm film, the film remains predominantly oxidized, and the retained gate-dependent transfer behavior argues against a continuous metallic source–drain shunt as the dominant origin of the increased off-state current. The corresponding output characteristics further confirm improved carrier injection. In Figure 3b, the 2 nm BiO_*x*_-capped MoS_2_ FET exhibits a higher drain current and a more linear low-field output behavior than the pristine device at L_CH_ = 100 nm and W_CH_ = 2 μm. Because BiO_*x*_ is deposited after formation of the Au electrodes, it does not directly access the buried Au/MoS_2_ interface underneath the metal. Instead, the improved output characteristics are primarily attributed to charge-transfer doping of the exposed contact-adjacent/access-region MoS_2_. The resulting increase in local electron density facilitates carrier injection and current spreading near the contact edge, thereby reducing the effective contact/access resistance.^33^ The statistical results in Figure 3c further illustrate the BiO_*x*_-induced threshold-voltage and current modulation. The extracted V_TH_ values show a systematic negative shift after 2 nm BiO_*x*_ capping. The drain-current statistics at V_BG_ = 4 V are retained as a common fixed-bias comparison among the pristine, 2 nm, and 6 nm BiO_*x*_ conditions to illustrate the overall thickness-dependent electrical modulation. Because the current measured at a fixed gate bias is strongly influenced by the accompanying V_TH_ shift, the pristine and 2 nm BiO_*x*_-capped devices were additionally compared at the same gate overdrive. At V_G_ – V_TH_ = 1 V, corresponding to n_2D_ = 1.11 × 10^13^ cm^–2^, the mean drain currents are 180.8 ± 12.6 and 185.6 ± 7.6 μA/μm for the pristine and 2 nm BiO_*x*_-capped devices, respectively, showing comparable current levels under equal carrier density. The corresponding equal-overdrive characteristics are provided in Figure S8.

**Figure 3:**
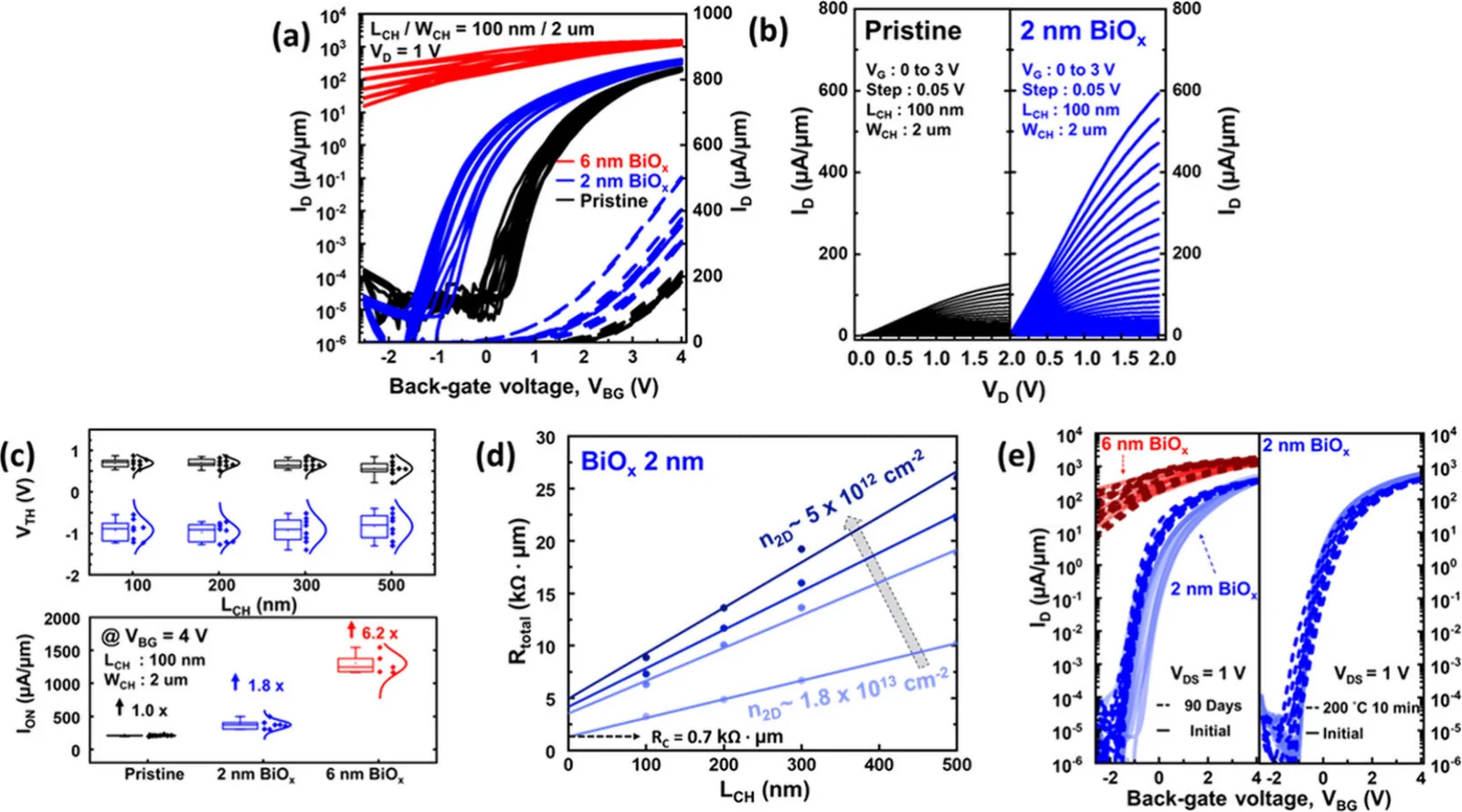
Device characteristics and stability of monolayer MoS_2_ FETs with BiO_*x*_ doping layers. Devices in panels (a) and (b) have L_CH_ = 100 nm and W_CH_ = 2 μm. (a) Transfer characteristics of MoS_2_ FETs before and after 2 and 6 nm BiO_*x*_ capping, measured at V_D_ = 1 V. Eight independently measured devices were analyzed for each condition (*n* = 8 per condition), with each data point representing one device. (b) Output characteristics of pristine and 2 nm BiO_*x*_-capped MoS_2_ FETs. The black curves represent the pristine MoS_2_ FET, while the blue curves represent the 2 nm BiO_*x*_-capped MoS_2_ FET. (c) Upper panel: extracted V_TH_ as a function of channel length for pristine and 2 nm BiO_*x*_-capped MoS_2_ FETs. Lower panel: statistical comparison of drain current measured at a common fixed gate bias of V_BG_ = 4 V for pristine, 2 nm BiO_*x*_-capped, and 6 nm BiO_*x*_-capped devices, illustrating the thickness-dependent electrical modulation. Because the fixed-bias current comparison includes the contribution of the V_TH_ shift, an additional equal-overdrive comparison between pristine and 2 nm BiO_*x*_-capped devices is provided in Figure S8. (d) Transfer length method analysis of 2 nm BiO_*x*_-capped MoS_2_ FETs. The total resistance is plotted as a function of channel length at different two-dimensional carrier densities, yielding R_C_ = 0.71 ± 0.06 kΩ·μm at n^2D^ = 1.8 × 10^13^ cm^–2^. Detailed device-to-device statistics, fitting uncertainties, and sheet-resistance extraction at n_2D_ = 1.8 × 10^13^ cm^–2^ are provided in Figure S7 and Table S3. All carrier-density-dependent TLM fits exhibit R^2^ ≈ 0.99. (e) Ambient stability characteristics of BiO_*x*_-capped MoS_2_ FETs. Left panel: transfer characteristics of 2 and 6 nm BiO_*x*_-capped devices before and after 90 days of air storage. Right panel: transfer characteristics of a 2 nm BiO_*x*_-capped device before and after 200 °C annealing in air for 10 min.

To quantify the effective contact resistance, TLM analysis was performed on 2 nm BiO_*x*_-capped MoS_2_ FETs with channel lengths of 100, 200, 300, and 500 nm, using eight independently measured devices at each channel length. At n_2D_ = 1.8 × 10^13^ cm^–2^, the analysis yields R_C_ = 0.71 ± 0.06 kΩ·μm and R_sh_ = 17.64 ± 0.36 kΩ/sq. Detailed TLM fitting, device-to-device statistics, and carrier-density calculations are provided in the Supporting Information. Here, the TLM-extracted R_C_ is interpreted as an effective contact resistance that includes contact-edge/junction and access-region contributions and should not be equated solely with the microscopic specific resistivity of the buried Au/MoS_2_ interface. The 6 nm BiO_*x*_ condition produces substantially stronger n-type modulation; however, this stronger doping is accompanied by a substantial increase in the off-state current, and the 6 nm BiO_*x*_-capped device cannot be fully depleted within the measured gate-voltage range. Therefore, the 6 nm condition represents a strongly doped regime rather than an optimized condition for transistor switching. In the present devices, 2 nm BiO_*x*_ provides a more favorable balance between contact-resistance reduction and gate controllability. The larger doping range accessible with thicker BiO_*x*_ may instead be useful for future selectively doped source/drain access or spacer regions, where a high local carrier density is desirable for reducing parasitic contact/access resistance while preserving electrostatic control of the central channel. A broader comparison with previously reported contact-engineering approaches is provided in Figure S2 of the Supporting Information. In addition to enhancing carrier injection, BiO_*x*_ also stabilizes the doped surface under ambient exposure.^16^ The stability measurements in Figure 3e were conducted under ambient conditions rather than high vacuum to evaluate the passivation function of the BiO_*x*_ overlayer. The transfer characteristics remain stable after 90 days in air and after 200 °C annealing in air for 10 min, indicating that the BiO_*x*_-induced doping/passivation effect is not transient.

To determine whether the same CTD strategy is transferable beyond MoS_2_, BiO_*x*_ was applied to monolayer WSe_2_ transistors. This extension is important because WSe_2_ is a central material for homogeneous TMD logic: its ambipolar character can support both n-type and p-type operation, but useful inverter behavior requires a sufficiently strong and well-controlled n-branch. Figure 4a shows the transfer characteristics of WSe_2_ FETs before and after 2 and 6 nm BiO_*x*_ capping, revealing a pronounced enhancement of the electron branch. Notably, BiO_*x*_ capping also suppresses the hole branch of the initially ambipolar WSe_2_ FET. Extended transfer measurements toward negative gate bias (Figure S3) show no recovery of the p-branch even when the measurement range is extended by an additional 1 V beyond that in Figure 4a. Thus, BiO_*x*_ increases the electron–hole conduction asymmetry and drives WSe_2_ toward predominantly electron-dominated transport, which is favorable for defining the n-FET polarity in the homogeneous inverter. The corresponding output characteristics in Figure 4b confirm the improved n-type conduction after 2 nm BiO_*x*_ deposition. The upper panel of Figure 4c further shows a systematic negative V_TH_ shift across different channel lengths after 2 nm BiO_*x*_ capping, confirming that the n-type modulation is maintained over the investigated device dimensions. As summarized in the lower panel of Figure 4c, at a common fixed gate bias of V_BG_ = 4 V, the drain-current ratios relative to the pristine condition are approximately 170 and 1390 for the 2 and 6 nm BiO_*x*_ conditions, respectively. Because these fixed-bias current ratios include the contribution of the BiO_*x*_-induced V_TH_ shift, they are used here to illustrate the overall thickness-dependent electrical modulation rather than an equal-carrier-density performance enhancement. XPS was used to examine the chemical states of WSe_2_ before and after BiO_*x*_ deposition, as shown in Figure 4d. The pristine WSe_2_ spectra exhibit W *4f* and Se *3d* peaks characteristic of W–Se bonding, whereas the BiO_*x*_-capped sample shows core-level shifts toward higher binding energy without evidence of severe chemical decomposition. These shifts are consistent with interfacial charge transfer and the associated Fermi-level movement induced by the BiO_*x*_ layer. UPS measurements of WSe_2_ were performed using the same inverted backside-probing geometry. As shown in Figure 4e, the secondary-electron cutoff values are approximately 16.80, 16.95, and 17.05 eV for samples prepared with nominal 2, 4, and 6 nm Bi, corresponding to TMD-side effective work functions of approximately 4.40, 4.25, and 4.15 eV, respectively. Because the exposed photoemission surface is monolayer WSe_2_ rather than the BiO_*x*_ overlayer, these thickness-dependent changes reflect the electronic modulation at the WSe_2_ side of the equilibrated BiO_*x*_/WSe_2_ heterostructure. The extracted E_F_ – E_V_ values simultaneously increase from approximately 1.00 to 1.15 and 1.25 eV with increasing nominal Bi thickness. Considering the literature-reported electronic bandgap of approximately 1.75 eV for monolayer WSe_2_ on Au,^34^ these results indicate that the WSe_2_ Fermi level progressively moves toward the conduction-band edge. The corresponding band alignment is summarized in Figure 4f. This trend parallels the MoS_2_ results, indicating that BiO_*x*_-induced energy-level modulation is applicable to both sulfide and selenide TMD channels. The strengthened WSe_2_ electron branch subsequently provides the n-type component required for the homogeneous inverter discussed below.

**Figure 4:**
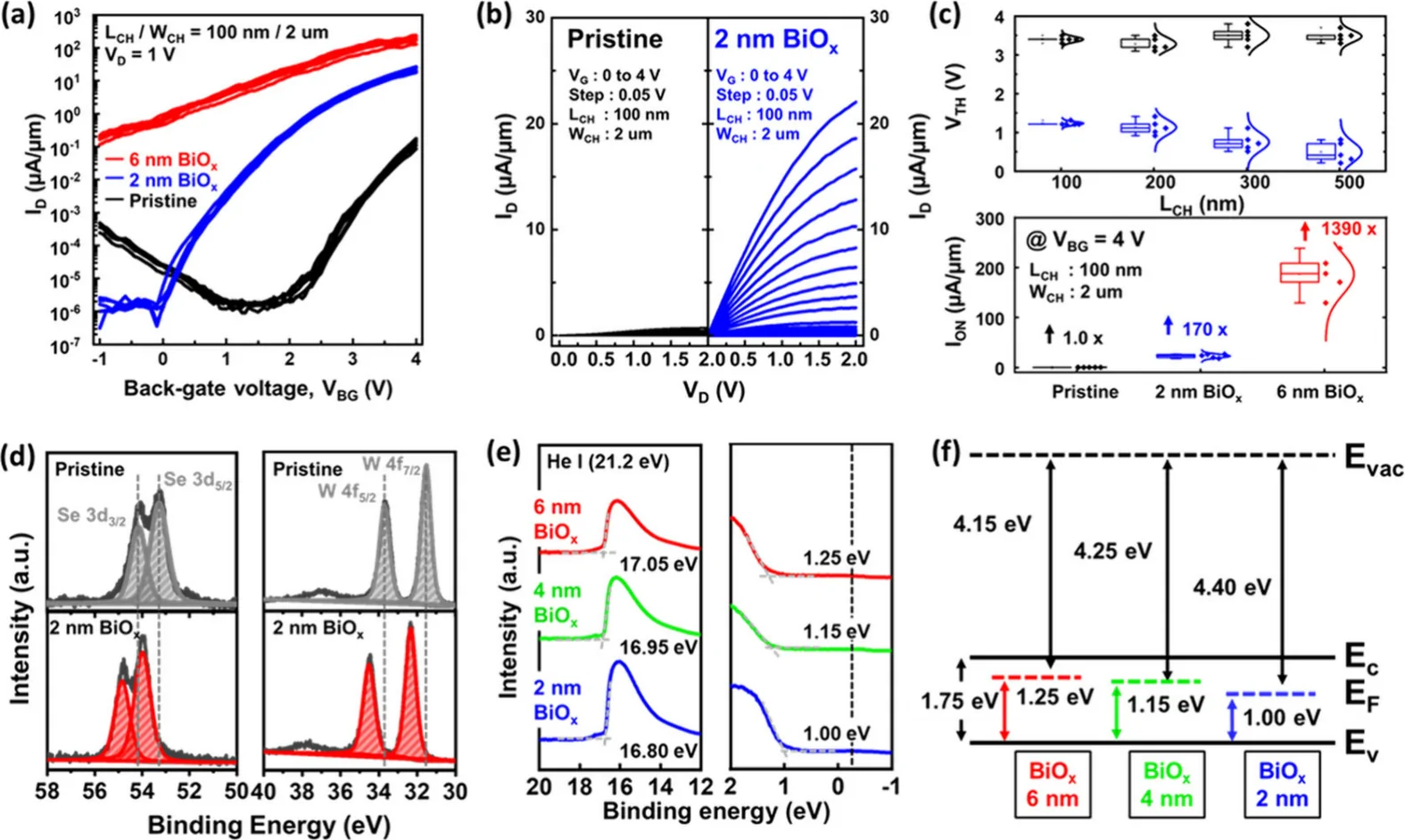
Extension of BiO_*x*_-induced n-type charge-transfer doping to monolayer WSe_2_ FETs. Devices in panels (a) and (b) have L_CH_ = 100 nm and W_CH_ = 2 μm. (a) Transfer characteristics of WSe_2_ FETs before and after 2 and 6 nm BiO_*x*_ capping. Eight independently measured WSe_2_ FETs were analyzed for each condition (*n* = 8 per condition). (b) Output characteristics of pristine and 2 nm BiO_*x*_-capped WSe_2_ FETs. The black curves represent the pristine WSe_2_ FET, while the blue curves represent the 2 nm BiO_*x*_-capped WSe_2_ FET. (c) Upper panel: extracted V_TH_ as a function of channel length for pristine and 2 nm BiO_*x*_-capped WSe_2_ FETs. Lower panel: fixed-bias drain-current comparison at V_BG_ = 4 V, showing current ratios of approximately 170 and 1390 relative to the pristine condition for the 2 and 6 nm BiO_*x*_ conditions, respectively. These fixed-bias ratios include the contribution of the BiO_*x*_-induced V_TH_ shift. (d) XPS spectra of Se *3d* and W *4f* core levels before and after 2 nm BiO_*x*_ deposition. (e) TMD-side UPS secondary-electron cutoff and valence-band spectra of backside-exposed BiO_*x*_/WSe_2_ heterostructures prepared with nominal 2, 4, and 6 nm Bi. The measurements were performed directly from the exposed WSe_2_ side after inverted transfer and removal of the sapphire growth substrate. (f) TMD-side energy-level alignment of the BiO_*x*_/WSe_2_ heterostructure derived from backside UPS measurements, showing progressive n-type Fermi-level modulation with increasing nominal Bi thickness. As for MoS_2_, the extracted values represent the WSe_2_-side electronic structure of the equilibrated BiO_*x*_/WSe_2_ heterostructure rather than the standalone work function of BiO_*x*_.

The functional relevance of BiO_*x*_-induced WSe_2_ n-type modulation was further evaluated using homogeneous WSe_2_ inverters. As illustrated in Figure 5a, the inverter is constructed on the same 1L-WSe_2_ film and HfO_*x*_/TiN gate stack, with a BiO_*x*_-doped WSe_2_ n-FET serving as the pull-down branch and a MoO_*x*_-doped WSe_2_ p-FET serving as the pull-up branch. The MoO_*x*_ layer enhances hole accumulation in the p-type branch, while the selectively patterned BiO_*x*_ layer strengthens electron transport in the n-type branch.^14,35–37^ The transfer characteristics and gate-leakage currents of the individual n-FET and p-FET are provided in Figure S4 of the Supporting Information. The gate leakage currents of both branches remain at approximately 10^–13^–10^–11^ A/μm, suggesting that gate-dielectric leakage is unlikely to be a major component of the inverter static power consumption. The output characteristics in Figure 5b show that the BiO_*x*_-engineered n-FET and MoO_*x*_-doped p-FET exhibit reasonably matched current levels, which is essential for obtaining a balanced voltage transfer characteristic. As shown in Figure 5c, the resulting homogeneous WSe_2_ inverter operates at V_DD_ = 2 V with a switching voltage close to V_M_ = 1 V, indicating that the pull-down and pull-up strengths are well balanced near the middle of the supply voltage. The corresponding voltage-gain curve in Figure 5c shows a maximum gain of approximately 19 near the transition point. Figure 5d further evaluates the inverter robustness using a butterfly plot, from which the noise margins are extracted as NM_L_ ≈ 0.45 V_DD_ and NM_H_ ≈ 0.445 V_DD_, giving a total normalized noise margin of 0.895 V_DD_. To evaluate circuit-level reproducibility, three independently fabricated homogeneous WSe_2_ inverters were characterized under the same operating conditions. All three devices exhibit complementary voltage-transfer behavior, as summarized in Figure S5, confirming that the inverter operation is reproducible across multiple independently fabricated circuits. The corresponding variations in maximum voltage gain, switching voltage V_M_, and normalized switching voltage V_M_/V_DD_ are summarized in Table S2.

**Figure 5:**
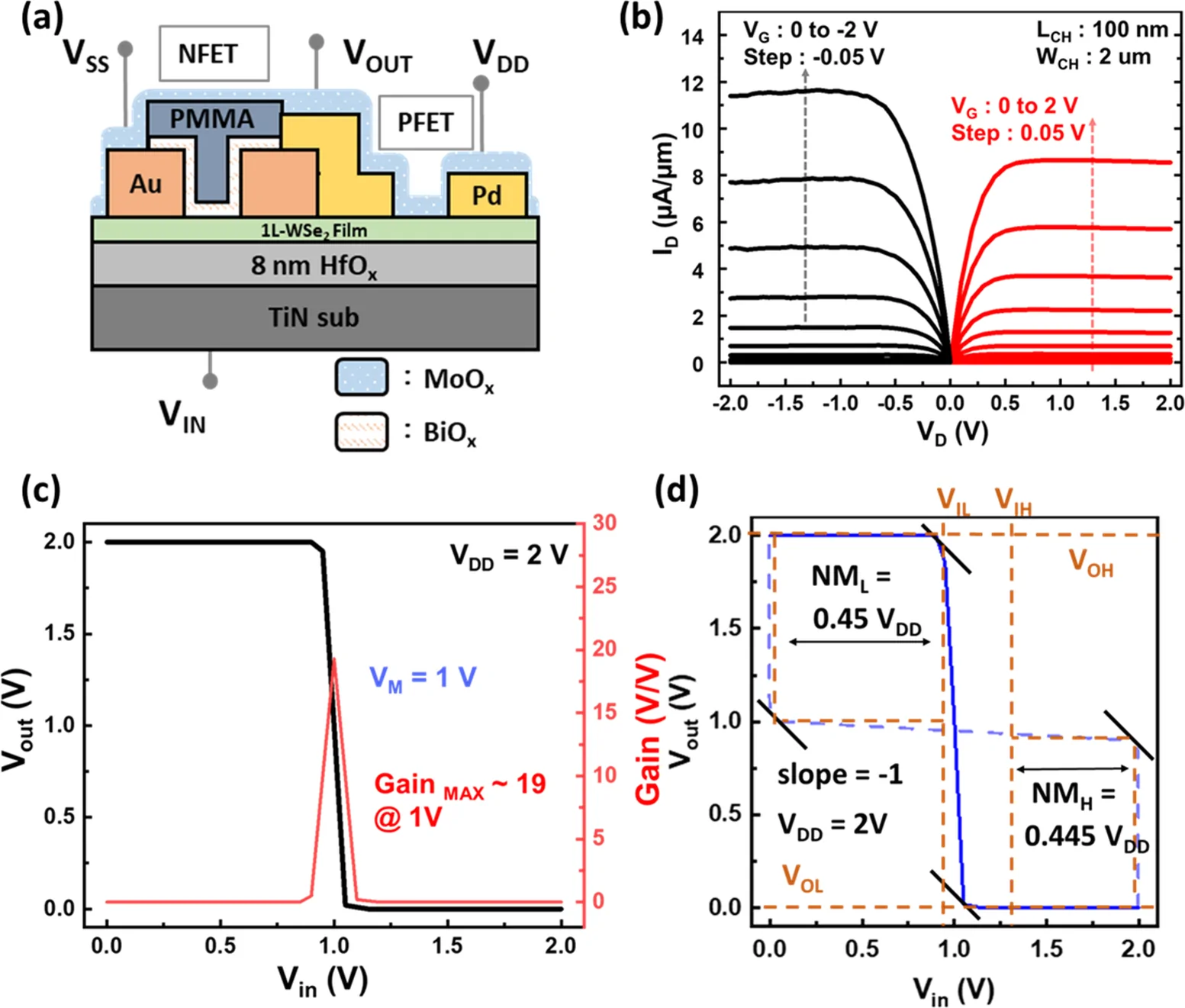
Homogeneous WSe_2_ inverter enabled by selective BiO_*x*_ and MoO_*x*_ doping. (a) Schematic illustration of the homogeneous WSe_2_ inverter consisting of a BiO_*x*_-doped n-FET pull-down branch and a MoO_*x*_-doped p-FET pull-up branch on an 8 nm HfO_*x*_/TiN gate stack. The n-FET uses Au contacts with selective BiO_*x*_ n-type doping, while the p-FET uses Pd contacts with selective MoO_*x*_ p-type doping. (b) Output characteristics of the BiO_*x*_-engineered WSe_2_ n-FET and MoO_*x*_-doped WSe_2_ p-FET. (c) Voltage transfer characteristic and corresponding voltage gain of the BiO_*x*_-engineered WSe_2_ homogeneous inverter at V_DD_ = 2 V. The inverter exhibits V_M_ ≈ 1 V and a maximum gain of approximately 19 near the switching point. (d) Butterfly plot for noise-margin extraction. The extracted noise margins are NM_L_ ≈ 0.45 V_DD_ and NM_H_ ≈ 0.445 V_DD_.

In conclusion, we demonstrate ultrathin BiO_*x*_ as a selectively patterned n-type charge-transfer doping layer for monolayer TMD devices. In MoS_2_ FETs, spectroscopic analyses confirm preserved channel integrity and thickness-dependent Fermi-level modulation, while electrical measurements yield a TLM-extracted effective contact resistance of 0.71 ± 0.06 kΩ·μm for 2 nm BiO_*x*_ at n_2D_ = 1.8 × 10^13^ cm^–2^. Increasing the BiO_*x*_ thickness produces stronger n-type modulation, while the 2 nm condition provides a more favorable balance between contact-resistance reduction and gate controllability. The doped devices also retain stable transfer characteristics after ambient storage and air annealing. Extending this approach to WSe_2_ enables strong electron-branch enhancement and balanced homogeneous inverter operation when paired with MoO_*x*_-based p-doping, achieving a gain of approximately 19 and a total normalized noise margin of 0.895 V_DD_. The combined transistor and inverter results identify BiO_*x*_ as a selectively patternable n-type interfacial modifier for monolayer TMD device integration.

## Supplementary Material



## Notes

Experimental details for device fabrication, BiO_*x*_ and MoO_*x*_ deposition, spectroscopic characterization, backside XPS/UPS measurements, and TLM/carrier-density analysis; thickness-dependent Bi *4f*/S *2p* and O *1s* XPS spectra and XPS-derived O/Bi atomic ratios of ambient-oxidized BiO_*x*_; contact-resistance benchmark of BiO_*x*_-capped monolayer MoS_2_ FETs; extended transfer characteristics of pristine and BiO_*x*_-capped monolayer WSe_2_ FETs; electrical characteristics of the individual WSe_2_ n-FET and p-FET and homogeneous WSe_2_ inverters; device-level TLM statistics and fitting uncertainties; and equal-gate-overdrive comparison of pristine and 2 nm BiO_*x*_-capped MoS_2_ FETs (PDF)
