# Supplementary material for: Ultrathin BiO x Charge-Transfer Doping for Cross-TMD Electronic Modulation and Homogeneous WSe2 Inverters
Source: Nano Lett. 2026 Oct 19;26(42):14675–82. doi: 10.1021/acs.nanolett.6c03585 (PMC13721172; doi:10.1021/acs.nanolett.6c03585)
Supplement: Supplementary file 1 [file nl6c03585_si_001.pdf]

Supporting information

Ultrathin BiO_x Charge-Transfer Doping for Cross-TMD Electronic Modulation and Homogeneous WSe_2 Inverters

Ching-Hao Hsu,[§∥] Yu-Wei Hsu,[¶∥] Ta-Wei Hsu,[§] Yu-Tung Lin,[§] Li-Ya Chiu,[§] I-Chih Ni,[§]

and Chih-I Wu,[§]*

[§]Graduate Institute of Photonics and Optoelectronics, National Taiwan University, Taipei 106, Taiwan

[¶]Graduate School of Advanced Technology, National Taiwan University, Taipei 106, Taiwan

Corresponding Author

Chih-I Wu — Email: chihiwu@ntu.edu.tw

Author Contributions

[∥]Ching-Hao Hsu and Yu-Wei Hsu contributed equally.

Methods

Fabrication of 1L-MoS_2 and 1L-WSe_2 FETs with BiO_x Doping Layers:

Monolayer MoS_2 and WSe_2 field-effect transistors were fabricated on TiN/8 nm HfO_x back-gate substrates. CVD-grown monolayer films were transferred onto the substrates and subsequently annealed at 200 °C under Ar flow to reduce transfer residues. Source/drain regions were defined by electron-beam lithography, followed by metal deposition and lift-off. Au contacts were used for the n-type MoS_2 and WSe_2 FETs. Ultrathin Bi layers with nominal thicknesses of 2, 4, and 6 nm were deposited by electron-beam evaporation using a lift-off-defined capping process. During deposition, a high-energy electron beam was used to heat and evaporate a Bi metal target, and the evaporated Bi species subsequently condensed on the TMD surface. The deposition was carried out at a base pressure of approximately 10^{-6} Torr and a rate of 0.01 Å/s. After deposition, the Bi-coated samples were exposed to laboratory ambient at room temperature for approximately 24 h before subsequent measurements, allowing the ultrathin Bi layers to undergo spontaneous oxidation and form BiO_x. No intentional thermal, plasma, ozone, or oxygen-assisted oxidation treatment was applied during this oxidation process, and the ambient relative humidity was not intentionally controlled. The oxidized Bi chemical states were subsequently verified by XPS. To avoid ambiguity, the stated BiO_x thicknesses refer to the nominal thicknesses of the evaporated Bi precursor layers prior to spontaneous ambient oxidation, unless otherwise specified.

Fabrication of Homogeneous 1L-WSe_2 Inverters:

Homogeneous WSe_2 inverters were fabricated using monolayer WSe_2 on the same TiN/8 nm HfO_x gate stack. The n-FET and p-FET regions were patterned separately by electron-beam lithography. Au contacts were used for the WSe_2 n-FET branch, while Pd contacts were used for the WSe_2 p-FET branch. After transistor fabrication, BiO_x was selectively deposited on the n-FET region to induce n-type charge-transfer doping. For the p-FET branch, a nominal 10 nm MoO_x layer was selectively deposited on the WSe_2 channel using PMMA protection and electron-beam-defined openings. MoO_x was deposited by thermal evaporation using 99.98% purity MoO_3 powder as the evaporation source. Prior to deposition, the chamber was pumped overnight to a base pressure of approximately 1.1×10^{-6} Torr. With the shutter closed, the MoO_3 source was pre-evaporated for approximately 5 min until the deposition rate stabilized at 0.5 Å/s. The shutter was then opened, and the MoO_x layer was deposited at 0.5 Å/s, with the chamber pressure maintained at approximately 2–3×10^{-6} Torr during deposition. This process produced a homogeneous WSe_2 inverter consisting of a BiO_x-doped n-FET pull-down branch and a MoO_x-doped p-FET pull-up branch.

Spectroscopic and Photoelectron Spectroscopy Measurements:

Raman and photoluminescence spectra were measured using a 473 nm blue laser

with a Kymera 328i spectrograph (Andor) to evaluate the structural and optical responses of the monolayer TMDs after BiO_x capping. X-ray photoelectron spectroscopy was performed using a ULVAC PHI 5000 Versa Probe III system with a monochromatic Al Kα source (hν = 1486.6 eV) to analyze the chemical states of the BiO_x-capped TMD surfaces. The measurements were conducted with a source power of 25 W, an accelerating voltage of 15 kV, and a beam spot size of 100 μm. High-resolution core-level spectra were collected using a pass energy of 27.00 eV and an energy step of 0.05 eV. Ultraviolet photoelectron spectroscopy was carried out on the same system using He I excitation (hν = 21.2 eV) to extract the secondary-electron cutoff, effective work function, and valence-band onset. For XPS and UPS characterization of the BiO_x/TMD interface, an inverted transfer configuration was employed to directly expose the TMD backside. As shown in Figure S6, monolayer MoS_2 and WSe_2 were first grown on sapphire substrates, followed by deposition of nominal Bi layers and spontaneous ambient oxidation to form BiO_x. The BiO_x side of the sample was then bonded to an Au-coated Si substrate using conductive Ag paste and allowed to fully dry. The sapphire growth substrate was subsequently removed, exposing the originally substrate-facing surface of the monolayer TMD. The resulting measurement geometry was vacuum/TMD/BiO_x/Ag/Au/Si. XPS and UPS measurements were therefore performed directly from the exposed TMD side

without sputter removal of the BiO_x layer.

TLM and Carrier-Density Analysis:

Transfer-length-method analysis was performed on 2 nm BiO_x-capped monolayer MoS_2 FETs with channel lengths of 100, 200, 300, and 500 nm. Eight independently measured devices were characterized at each channel length. The threshold voltage V_{TH} was individually extracted for each device by linear fitting of its transfer characteristic. The two-dimensional carrier density was calculated as $n_{2D}=C_{BG}(V_G-V_{TH})/q$, where $C_{BG}=\varepsilon_0\varepsilon_r/t_{HfOx}$. Using $\varepsilon_r=16$ and $t_{HfOx}=8$ nm gives $C_{BG}=1.77\times10^{-6}$ F/cm². For each device, the gate voltage was individually selected according to its extracted V_{TH} to achieve the same target carrier density. The width-normalized total resistance was calculated at $V_D=1$ V as $R_{tot}W=(V_D/I_D)W$ and analyzed according to $R_{tot}W=R_{sh}L_{CH}+2R_C$.

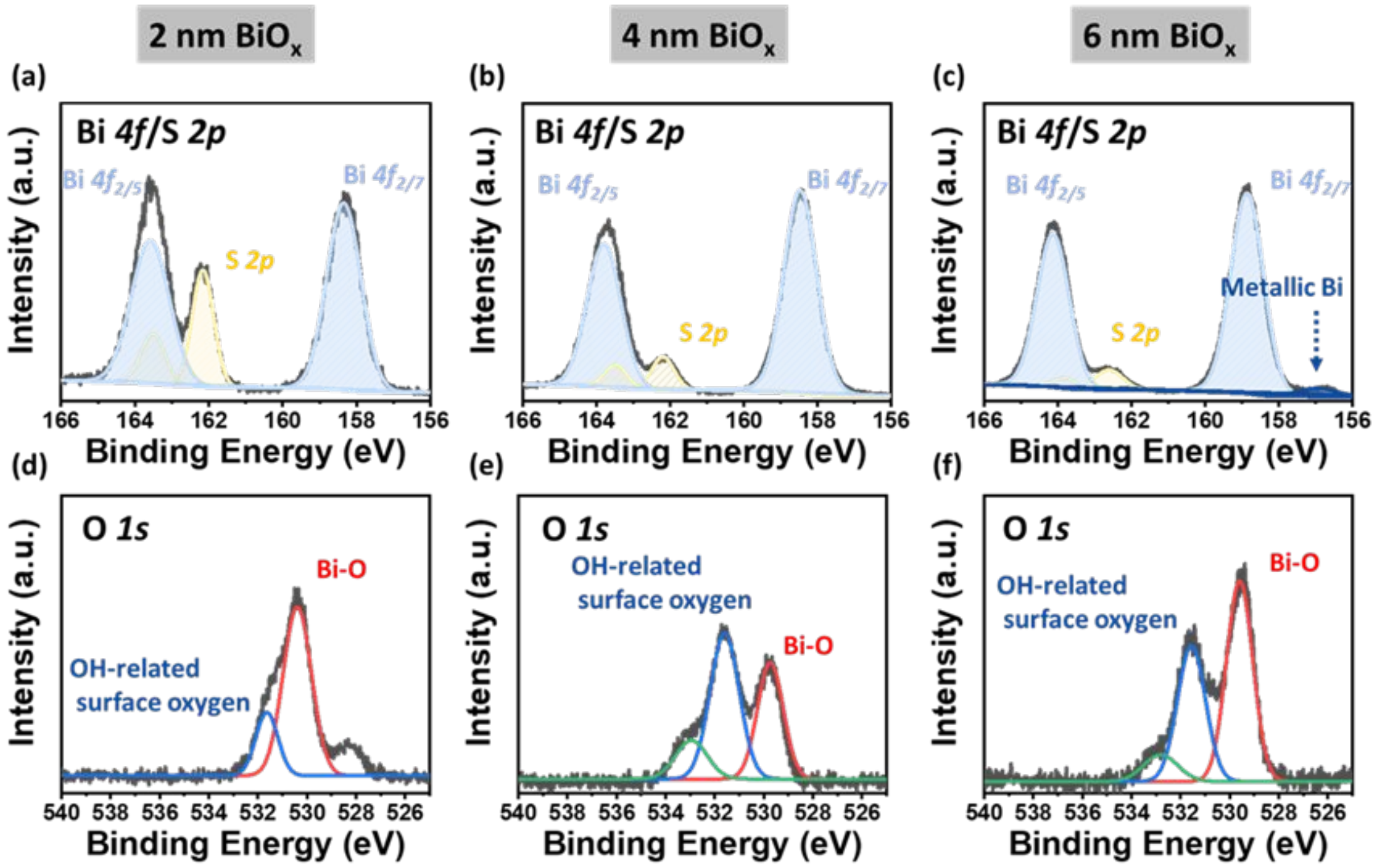

Figure S1. Thickness-dependent XPS analysis of ambient-oxidized BiO_x layers formed from nominally 2, 4, and 6 nm evaporated Bi.

Nominal Bi thickness	O/Bi atomic ratio	Bi:O atomic ratio	XPS interpretation
**2 nm**	**1.3**	**1:1.3**	**Substoichiometric BiO_x; no resolved Bi^0**
**4 nm**	**1.1**	**1:1.1**	**More oxygen-deficient BiO_x; no resolved Bi^0**
**6 nm**	**1.0**	**1:1.0**	**Most oxygen-deficient; minor residual Bi^0**

Table S1. XPS-derived atomic composition of ambient-oxidized BiO_x layers prepared from different nominal Bi thicknesses. (The reported compositions represent average XPS-derived O/Bi atomic ratios and should not be interpreted as assignments to specific crystalline bismuth-oxide phases.)

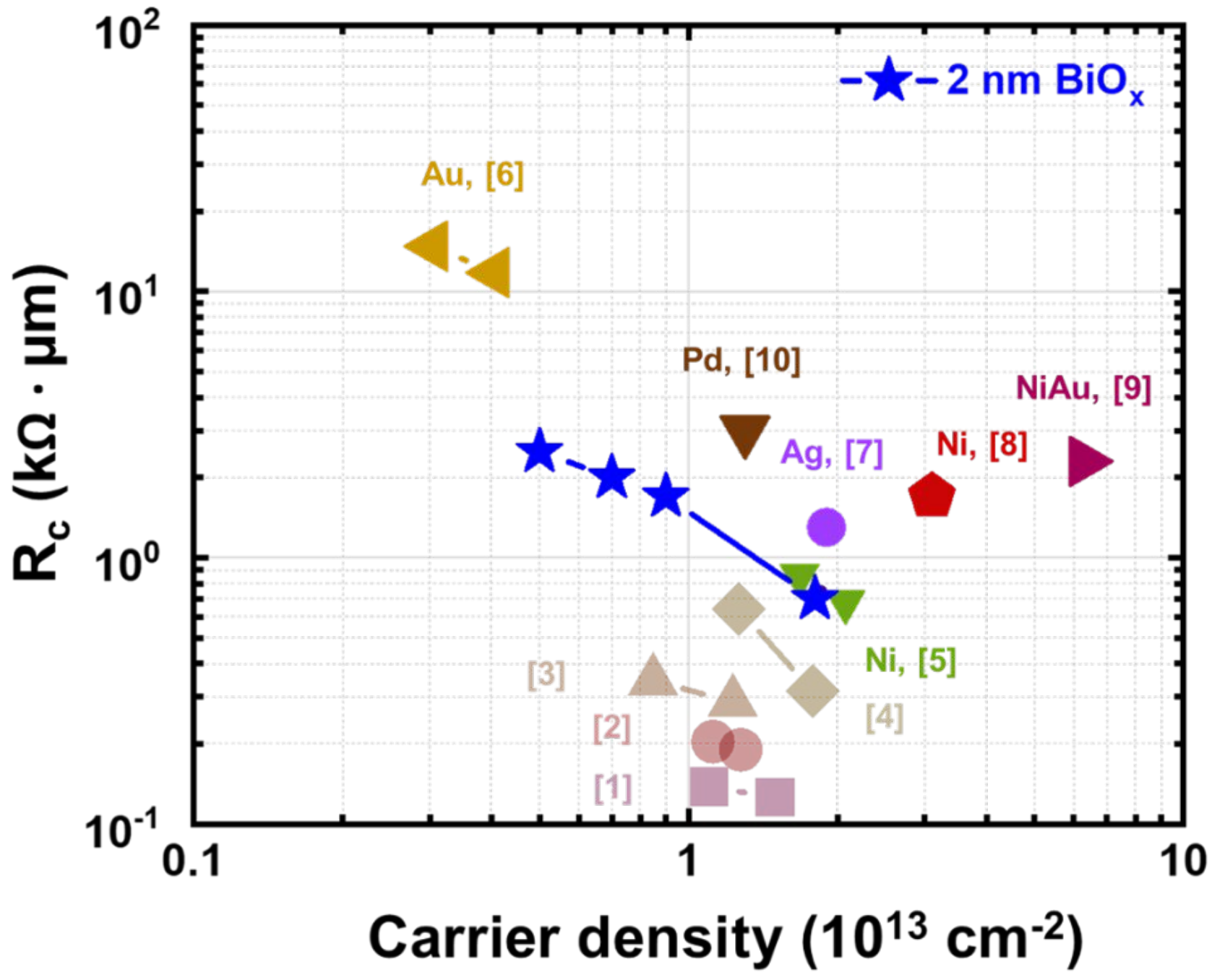

Figure S2. Contact-resistance benchmark of 2 nm BiO_x-capped monolayer MoS_2 FETs. Benchmark of the effective contact resistance R_C as a function of two-dimensional carrier density (n_{2D}) for 2 nm BiO_x-capped MoS_2 FETs and previously reported contact-engineering approaches. The blue stars represent the results obtained using 2 nm BiO_x. The semi-transparent symbols denote low-thermal-budget contact schemes, including semimetal contacts.

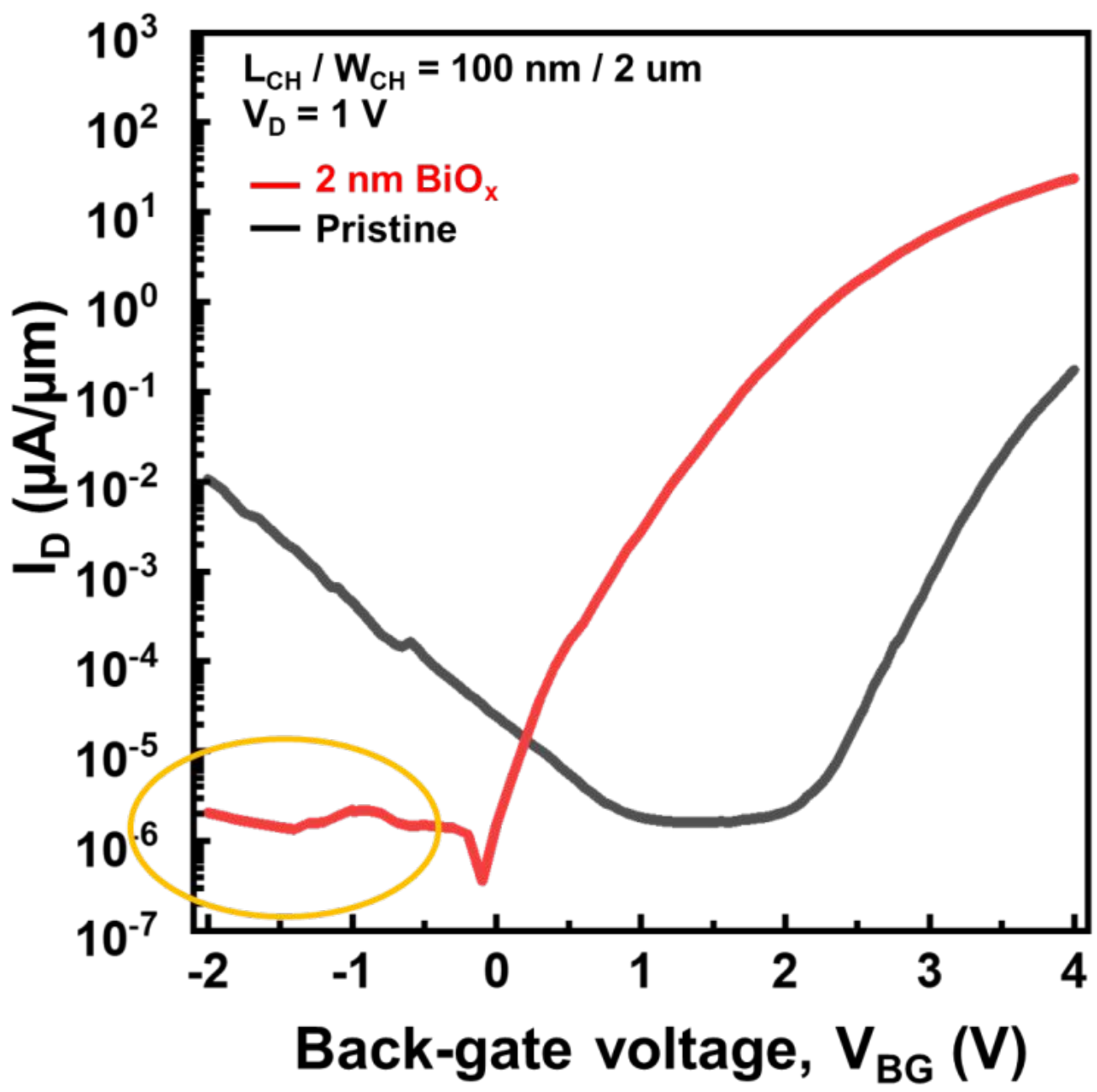

Figure S3. Extended transfer characteristics of pristine and 2 nm BiO_x-capped monolayer WSe_2 FETs.

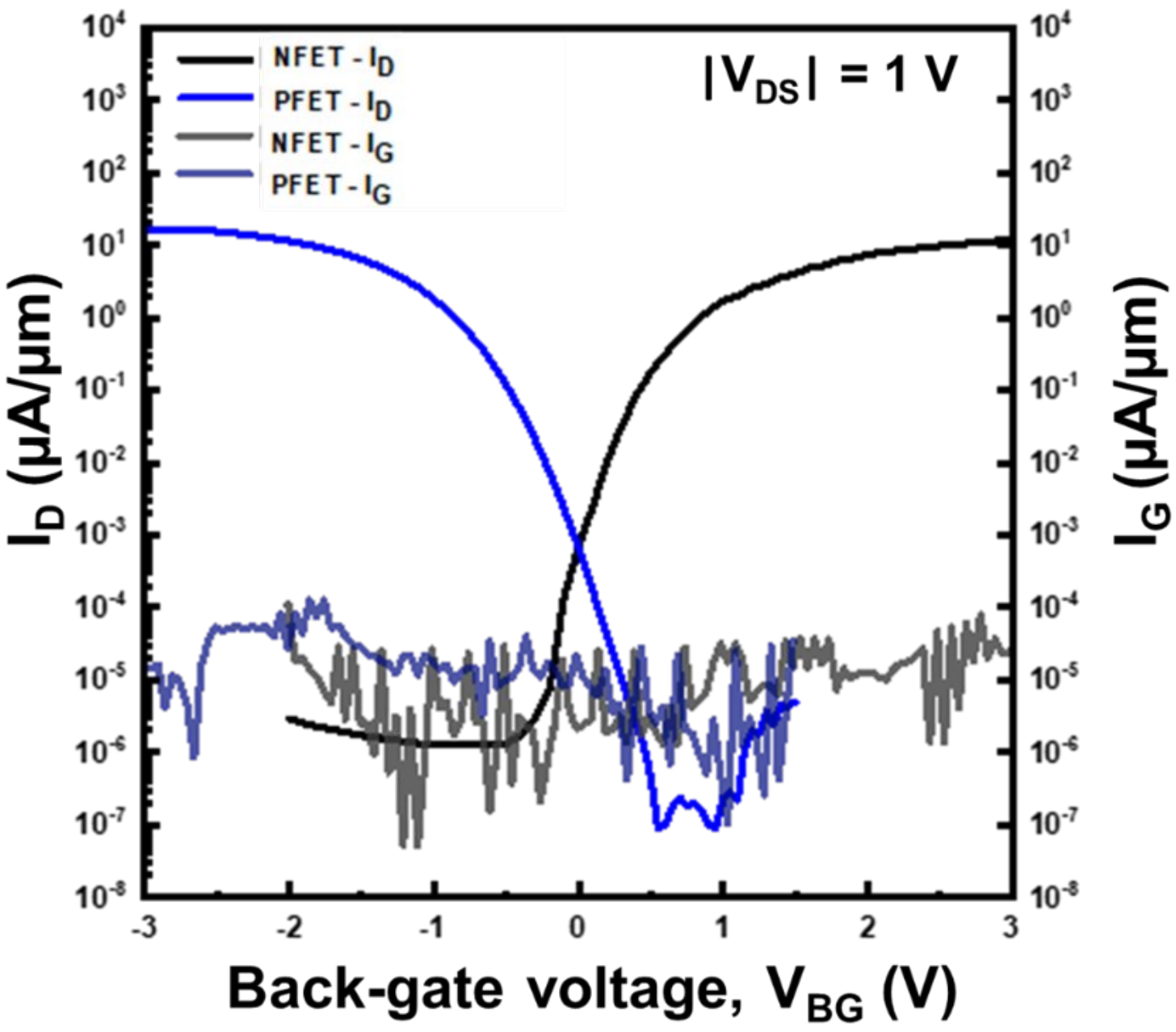

Figure S4. Transfer characteristics and gate-leakage currents of the individual WSe_2 transistors used in the homogeneous inverter. Drain-current characteristics (I_D) of the BiO_x-doped WSe_2 n-FET and MoO_x-doped WSe_2 p-FET are shown together with their corresponding gate-leakage currents (I_G). Both devices exhibit gate-leakage currents in the range of approximately 10^{-13}–10^{-11} A/μm.

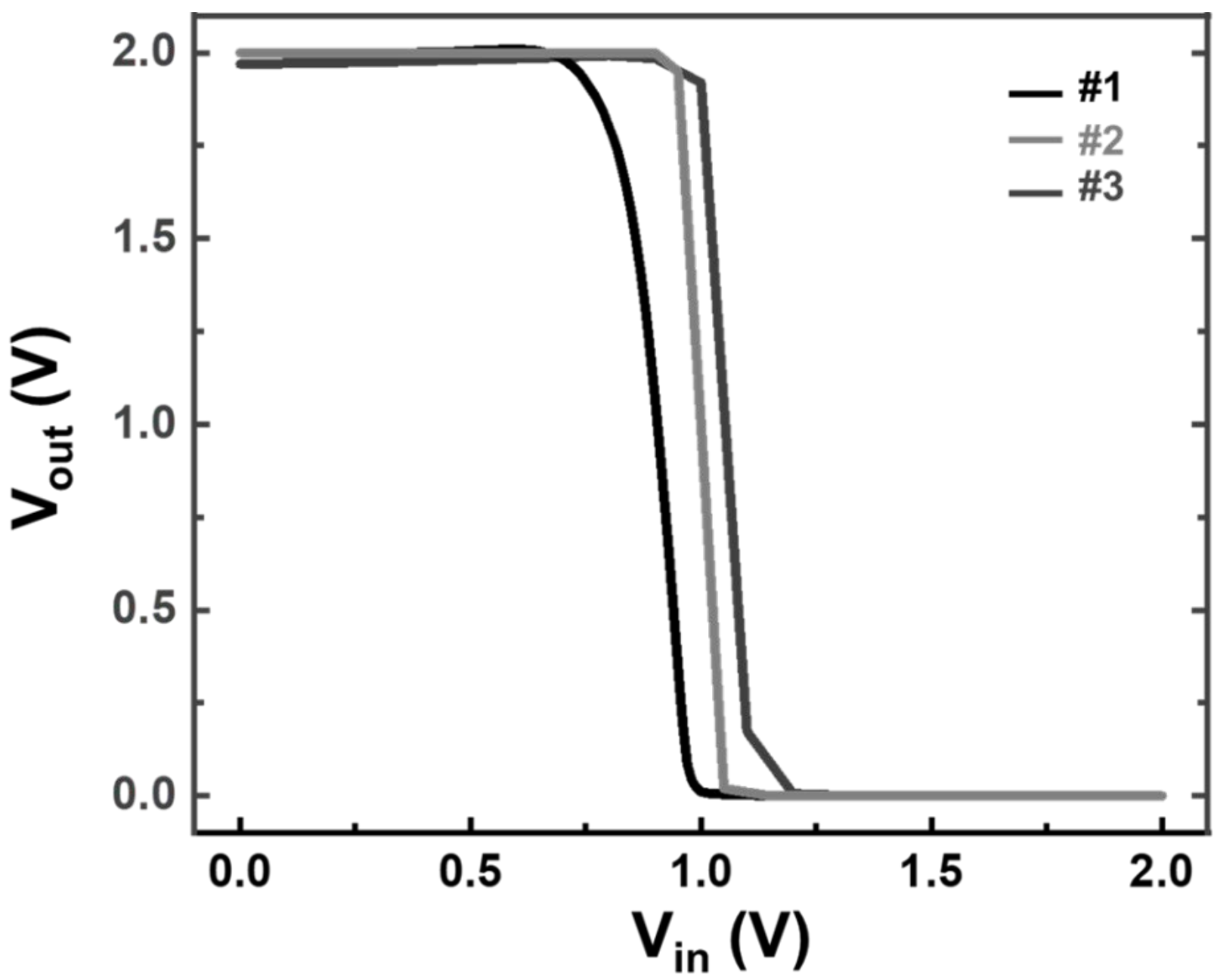

Figure S5. Device-to-device variation of homogeneous WSe_2 inverters.

Inverter	Voltage gain (V/V)	V_M (V)	V_M/V_{DD}
**#1**	**9.6**	**1.05**	**0.525**
**#2**	**15.5**	**0.91**	**0.455**
**#3**	**19.0**	**1.00**	**0.5**
**Mean ± SD**	**14.7 ± 4.8**	**0.99 ± 0.07**	**0.493 ± 0.035**

Table S2. Electrical characteristics of three independently fabricated homogeneous WSe_2 inverters measured at V_{DD} = 2 V.

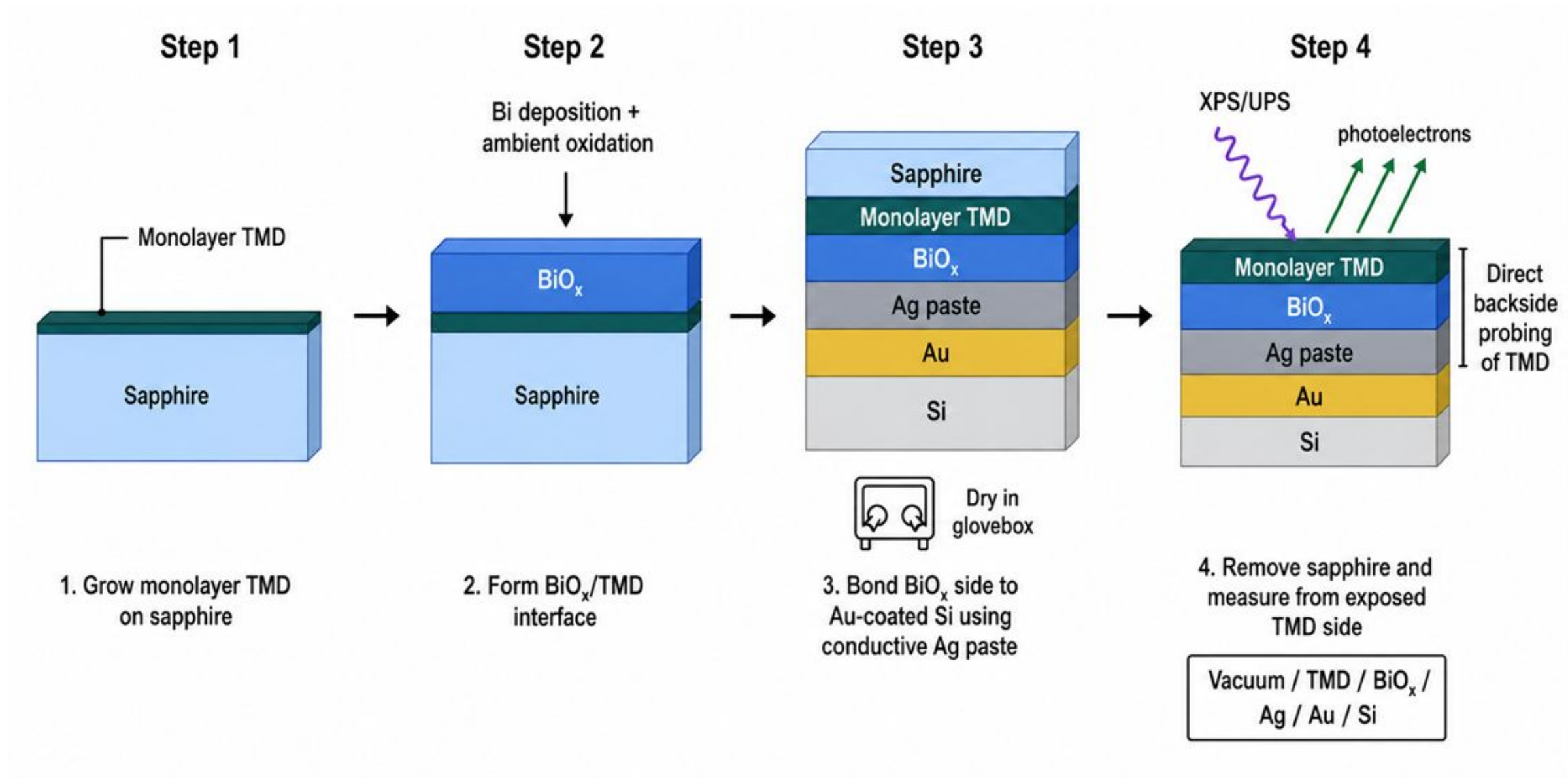

Figure S6. Sample-preparation procedure and measurement geometry for backside XPS/UPS characterization of BiO_x/TMD heterostructures.

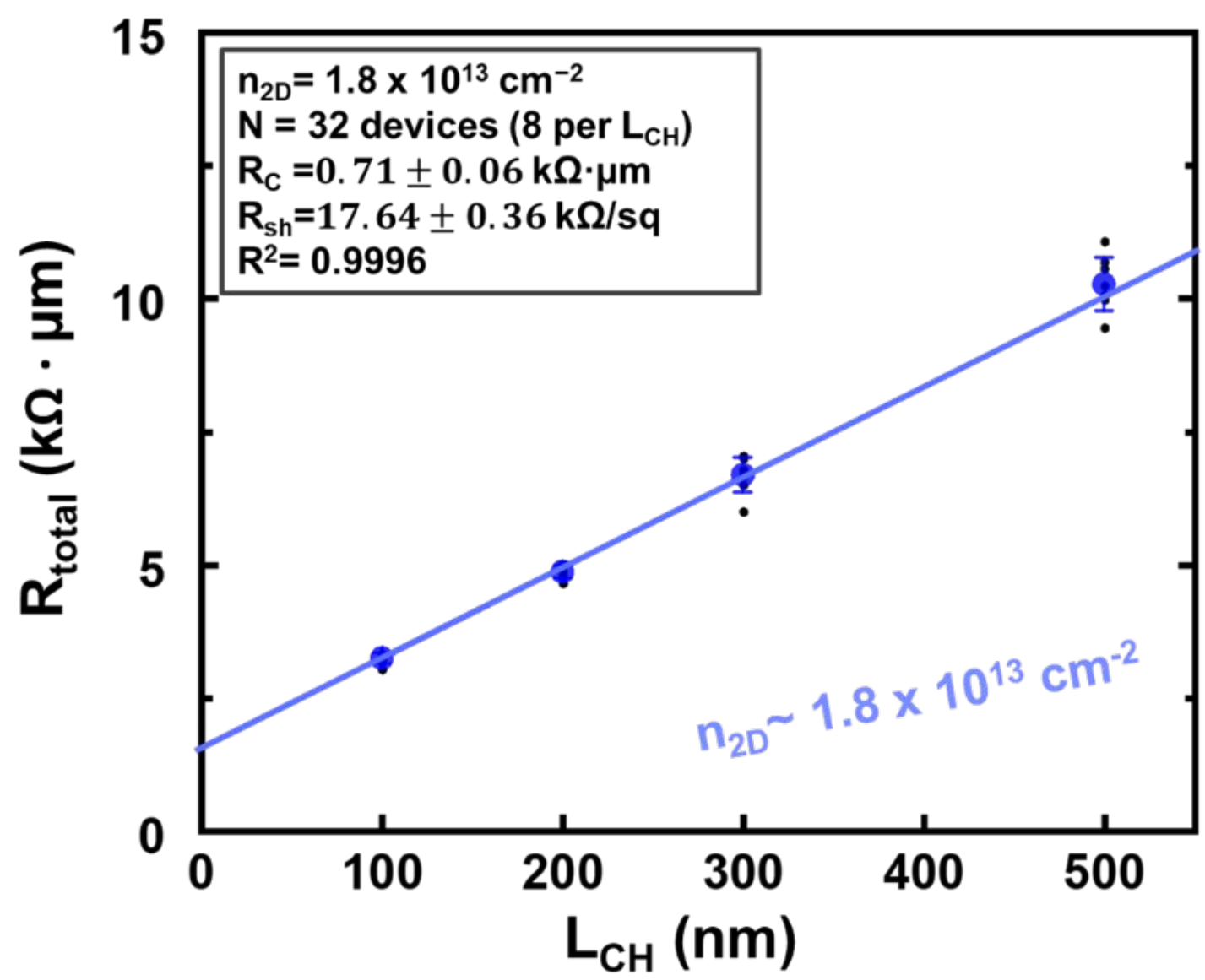

Figure S7. Device-level statistical analysis of the TLM measurement for 2 nm BiO_x-capped monolayer MoS_2 FETs at n_{2D}=1.8×10^{13} cm^{-2}. Eight independently measured devices were characterized at each channel length of 100, 200, 300, and 500 nm, corresponding to 32 devices in total. Small symbols represent the individual-device $R_{tot}W$ values, whereas the larger symbols and error bars represent the mean ± one standard deviation at each channel length. The solid line represents the mean-value TLM fit according to $R_{tot}W=R_{sh}L_{CH}+2R_C$, yielding R^2=0.9996. The extracted R_C and R_{sh} are 0.71±0.06 kΩ·µm and 17.64±0.36 kΩ/sq, respectively. The uncertainties in R_C and R_{sh} represent the standard errors obtained from regression analysis including the individual-device resistance values.

LCH (nm)	Number of devices	Mean RtotW (kΩ·µm)	SD (kΩ·µm)
100	8	3.243	0.102
200	8	4.863	0.123
300	8	6.685	0.327
500	8	10.262	0.5

n2D (cm^-2)	Total devices	RC (kΩ·µm)	Rsh (kΩ/sq)	Mean-value fit R^2
1.8×10^13	32	0.71 ± 0.06	17.64 ± 0.36	0.9996

Table S3. Device statistics and TLM fitting parameters for 2 nm BiO_x-capped monolayer MoS_2 FETs at $n^{2D}=1.8\times10^{13}$ cm^{-2}.

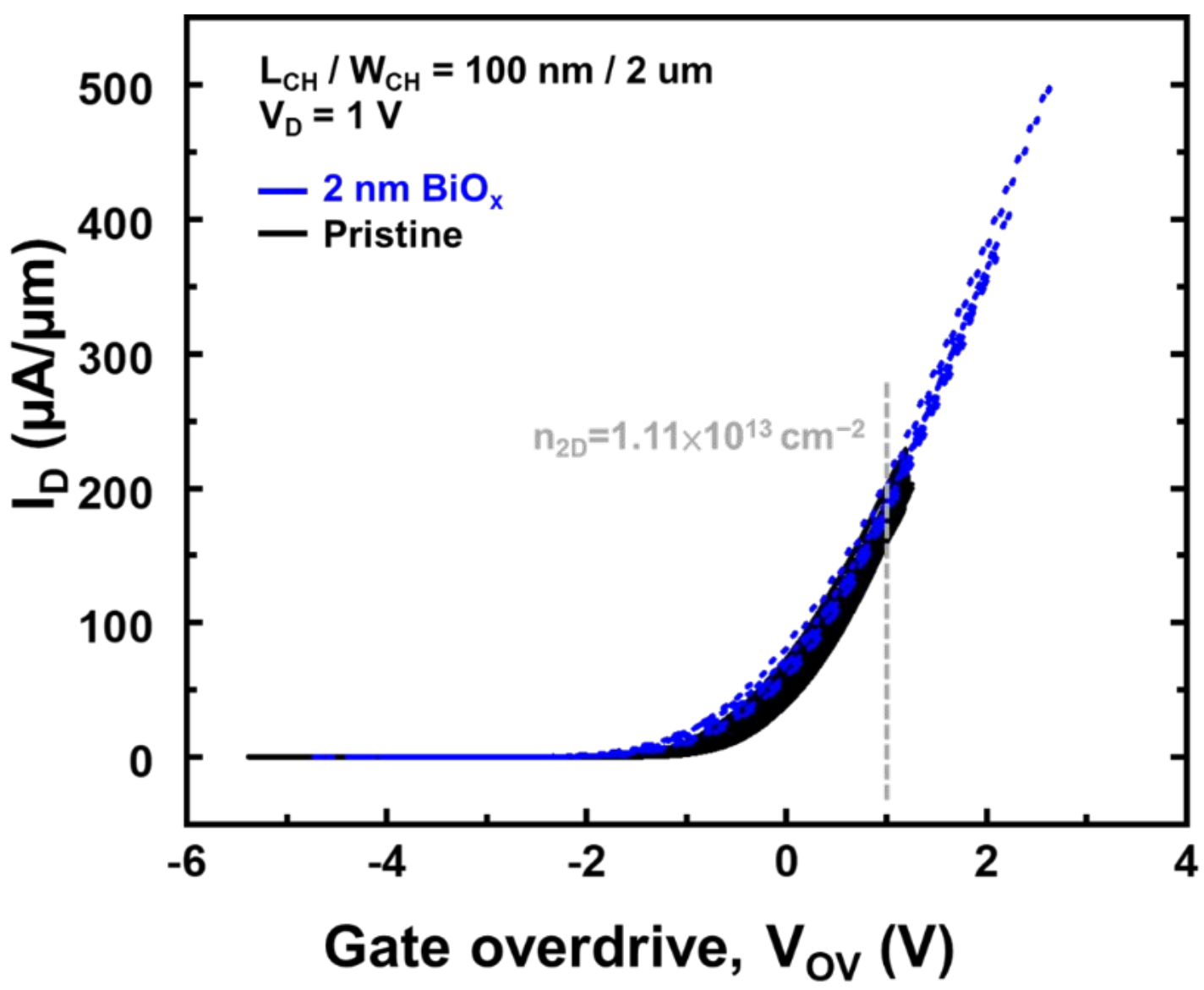

Figure S8. Equal-gate-overdrive comparison of pristine and 2 nm BiO_x-capped monolayer MoS_2 FETs. Transfer characteristics of eight independently measured pristine devices and eight independently measured 2 nm BiO_x-capped devices are replotted as a function of gate overdrive, $V_{OV}=V_G-V_{TH}$, at V_D=1 V. The dashed vertical line indicates V_{OV}=1 V, corresponding to n2D=1.11×10^{13} cm^{-2}. At this equal-carrier-density condition, the mean drain currents are 180.8±12.6 and 185.6±7.6 µA/µm for the pristine and 2 nm BiO_x-capped devices, respectively, where the uncertainties represent one standard deviation among the eight devices.

REFERENCES

(1) Shen, P.-C.; Su, C.; Lin, Y.; Chou, A.-S.; Cheng, C.-C.; Park, J.-H.; Chiu, M.-H.; Lu, A.-Y.; Tang, H.-L.; Tavakoli, M. M.; Pitner, G.; Ji, X.; Cai, Z.; Mao, N.; Wang, J.; Tung, V.; Li, J.; Bokor, J.; Zettl, A.; Wu, C.-I.; Palacios, T.; Li, L.-J.; Kong, J. Ultralow Contact Resistance between Semimetal and Monolayer Semiconductors. *Nature* **2021**, *593*, 211–217. **https://doi.org/10.1038/s41586-021-03472-9.**

(2) Wang, Y.; Kim, J. C.; Wu, R. J.; Martinez, J.; Song, X.; Yang, J.; Zhao, F.; Mkhoyan, K. A.; Jeong, H. Y.; Chhowalla, M. Van der Waals Contacts between Three-Dimensional Metals and Two-Dimensional Semiconductors. *Nature* **2019**, *568*, 70–74. **https://doi.org/10.1038/s41586-019-1052-3.**

(3) Kumar, A.; Schauble, K.; Neilson, K. M.; Tang, A.; Ramesh, P.; Wong, H.-S. P.; Pop, E.; Saraswat, K. Sub-200 Ω·µm Alloyed Contacts to Synthetic Monolayer MoS_2. In *2021 IEEE International Electron Devices Meeting (IEDM)*; IEEE, 2021; pp 7.3.1–7.3.4. **https://doi.org/10.1109/IEDM19574.2021.9720609.**

(4) Li, W.; Gong, X.; Yu, Z.; Ma, L.; Sun, W.; Gao, S.; Köroğlu, C.; Wang, W.; Liu, L.; Li, T.; Ning, H.; Fan, D.; Xu, Y.; Tu, X.; Xu, T.; Sun, L.; Wang, W.; Lu, J.; Ni, Z.; Li, J.; Duan, X.; Wang, P.; Nie, Y.; Qiu, H.; Shi, Y.; Pop, E.; Wang, J.; Wang, X. Approaching the Quantum Limit in Two-Dimensional Semiconductor Contacts. *Nature* **2023**, *613*, 274–279.

https://doi.org/10.1038/s41586-022-05520-0.

(5) Sun, Z.; Kim, S. Y.; Cai, J.; Shen, J.; Lan, H.-Y.; Tan, Y.; Wang, X.; Shen, C.; Wang, H.; Chen, Z.; Wallace, R. M.; Appenzeller, J. Low Contact Resistance on Monolayer MoS_2 Field-Effect Transistors Achieved by CMOS-Compatible Metal Contacts. *ACS Nano* **2024**, *18*, 22444–22453.

https://doi.org/10.1021/acsnano.4c07267.

(6) Smithe, K. K. H.; English, C. D.; Suryavanshi, S. V.; Pop, E. Intrinsic Electrical Transport and Performance Projections of Synthetic Monolayer MoS_2 Devices. *2D Mater.* **2017**, *4*, 011009.

https://doi.org/10.1088/2053-1583/4/1/011009.

(7) Smithe, K. K. H.; English, C. D.; Suryavanshi, S. V.; Pop, E. High-Field Transport and Velocity Saturation in Synthetic Monolayer MoS_2. *Nano Lett.* **2018**, *18*, 4516–4522. **https://doi.org/10.1021/acs.nanolett.8b01692.**

(8) Smets, Q.; Arutchelvan, G.; Jussot, J.; Verreck, D.; Asselberghs, I.; Mehta, A. N.; Gaur, A.; Lin, D.; El Kazzi, S.; Groven, B.; Caymax, M.; Radu, I. Ultra-Scaled MOCVD MoS_2 MOSFETs with 42 nm Contact Pitch and 250 µA/µm Drain Current. In *2019 IEEE International Electron Devices Meeting (IEDM)*; IEEE, 2019; pp 23.2.1–23.2.4. **https://doi.org/10.1109/IEDM19573.2019.8993650.**

(9) Zhu, Y.; Li, Y.; Arefe, G.; Burke, R. A.; Tan, C.; Hao, Y.; Liu, X.; Liu, X.; Yoo, W. J.; Dubey, M.; Lin, Q.; Hone, J. C. Monolayer Molybdenum Disulfide Transistors with Single-Atom-Thick Gates. *Nano Lett.* **2018**, *18*, 3807–3813. **https://doi.org/10.1021/acs.nanolett.8b01091.**

(10) Zheng, X.; Calò, A.; Albisetti, E.; Liu, X.; Alharbi, A. S. M.; Arefe, G.; Liu, X.; Spieser, M.; Yoo, W. J.; Taniguchi, T.; Watanabe, K.; Aruta, C.; Ciarrocchi, A.; Kis, A.; Lee, B. S.; Lipson, M.; Hone, J.; Shahrjerdi, D.; Riedo, E. Patterning Metal Contacts on Monolayer MoS_2 with Vanishing Schottky Barriers Using Thermal Nanolithography. *Nat. Electron.* **2019**, *2*, 17–25.

https://doi.org/10.1038/s41928-018-0191-0.
